# Comparação entre a Relação Neutrófilo-Linfócito Precoce e Tardia na Predição de Eventos Adversos em Pacientes com IAMCSST submetidos à ICP Primária

**DOI:** 10.36660/abc.20200327

**Published:** 2021-03-03

**Authors:** Guilherme Pinheiro Machado, Gustavo Neves de Araujo, Daniele Maltauro, Julia Custodio, Victoria Milan, Marco Wainstein

**Affiliations:** 1 Universidade Federal do Rio Grande do Sul Porto AlegreRS Brasil Universidade Federal do Rio Grande do Sul, Porto Alegre, RS - Brasil; 2 Hospital de Clínicas de Porto Alegre Porto AlegreRS Brasil Hospital de Clínicas de Porto Alegre, Porto Alegre, RS - Brasil; 3 Universidade Federal de Ciências da Saúde de Porto Alegre Faculdade de Medicina Porto AlegreRS Brasil Universidade Federal de Ciências da Saúde de Porto Alegre Faculdade de Medicina, Porto Alegre, RS - Brasil

**Keywords:** Infarto do Miocárdio, Angioplastia, Mortalidade, relação neutrófilo-linfócito, Inflamação, Fatores de Risco

## Introdução

A relação neutrófilo-linfócito (NLR) na admissão hospitalar já se mostrou capaz de prever eventos adversos em pacientes com infarto agudo do miocárdio com supra desnível do segmento ST (IAMCSST).1-3 Evidências recentes demonstraram que a NLR continua aumentando no período de 48 a 72 horas nos pacientes que apresentam resultados piores.4 Portanto, nosso objetivo foi comparar a capacidade prognóstica da NLR na admissão e tardia para eventos adversos em pacientes com IAMCSST submetidos à intervenção coronária percutânea primária (ICPp).

## Métodos

Este foi um estudo coorte prospectivo com pacientes consecutivos admitidos com IAMCSST que passaram por ICPp e foram acompanhados durante 12 meses. A NLR foi calculada dividindo-se o número de neutrófilos pelo número de linfócitos obtidos da mesma amostra sanguínea. A NLR foi avaliada na admissão e no período de 48 a 72 horas após o procedimento (NLR tardia) como parte do tratamento de rotina. Outros detalhes sobre informações procedimentais, coleta de dados, definições clínicas, critérios de exclusão e diretrizes éticas estão descritos em outros locais.^2^ A NLR alta foi definida como acima do tercil superior. A análise da curva de característica de operação do receptor (ROC) foi realizada para calcular a área sob a curva (AUC) para a ocorrência de mortalidade em curto e longo prazo e de eventos cardíacos adversos maiores (MACE) Foram realizadas análises multivariadas pela regressão de Poisson com variância robusta para avaliar o valor preditivo independente da NLR tardia. Para o modelo multivariado, os fatores de risco que foram preditores univariados (p <0,05) foram considerados inicialmente como fatores ou covariáveis. As análises de concordância foram comparadas pelo teste de De Long, enquanto os métodos de Kaplan-Meier foram comparados com testes de Log-Rank, realizados utilizando-se o software MedCalc Statistical, versão 14.8.1 (MedCalc Software, Ostend, Bélgica). Todas as demais análises estatísticas foram realizadas utilizando-se o software SPSS Statistics for Windows, v.21.0. (IBM Corp., Armonk, NewYork, EUA).

## Resultados

Entre março de 2011 e dezembro de 2018, 864 pacientes compareceram à nossa instituição, diagnosticado com IAMCSST, e 779 deles foram incluídos na análise. A média de idade foi de 60,68 (±12), 66,4% dos pacientes eram do sexo masculino, 62,1% tinham hipertensão e 24% tinham diabetes.

**Figura 1:**
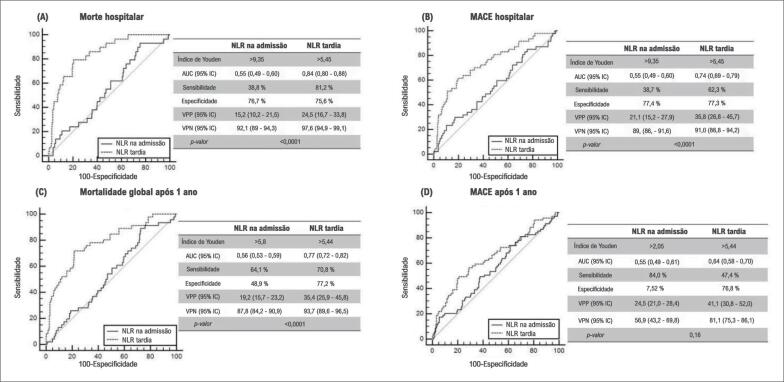
Gráfico de característica de operação do receptor (ROC) mostrando as áreas sob acurva (AUC) da relação neutrófilo-linfócito (NLR) na admissão e NLR tardia para (A) mortalidade hospitalar, (B) eventos cardiovasculares maiores hospitalares (MACE), (C) mortalidade global após 1 ano e (D) MACE após 1 ano.

**Figura 2:**
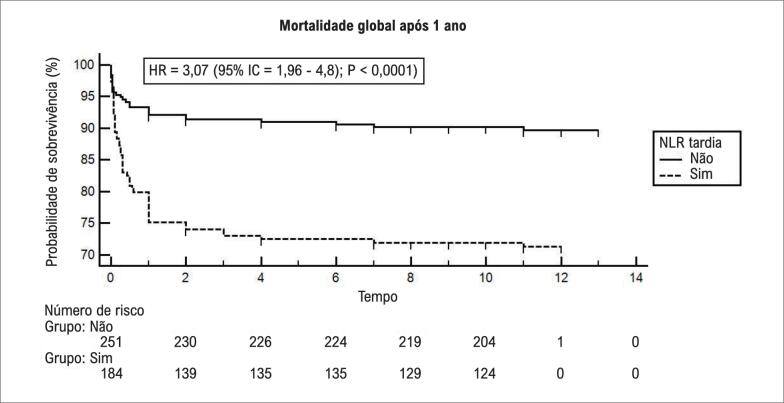
Curvas tempo-evento para mortalidade global após 1 ano de relação neutrófilo-linfócito (NLR). Os índices de eventos foram calculados utilizando-se os métodos de Kaplan-Meier, e comparados com uso do teste Log-rank

Na análise multivariada, quando ajustada por idade, tempo dor-porta, doença renal crônica prévia, infarto do miocárdio (IM) prévio, hipotensão na admissão, acesso femoral, tempo de fluoroscopia, volume de contraste, classificação de trombose no infarto do miocárdio (TIMI), fração de ejeção do ventrículo esquerdo ≤40% antes da alta, a NLR continuou sendo um preditor independente da mortalidade hospitalar, MACE hospitalar e mortalidade após 1 ano (risco relativo [RR] = 14,9, 95% intervalo de confiança [95% IC]= 3,4 - 80,35, p= 0,001; RR= 3,4, 95% IC= 1,2 – 9,1, p= 0,01; RR= 7,6, 95% IC= 2,9 – 26,1, p= 0,01, respectivamente). O uso da NLR tardia aumentou significativamente a AUC de mortalidade hospitalar de 0,55 para 0,84 (Sensibilidade 81,2%, Especificidade 75,6%, Valor preditivo positivo 24,5 e Valor preditivo negativo 97,7). Os dados discriminados dos demais resultados estão descritos na Figura 1. Ao final de 1 ano de acompanhamento, o índice de mortalidade global foi de 28,6% no grupo de NLR alta, hazard ratio (HR) = 3,07 (95% IC= 1,9 - 4,8); p< 0,0001; Figura 2).

## Discussão

No presente estudo coorte de pacientes de IAMCSST que passaram por ICPp, a NLR tardia foi fortemente associada à mortalidade no curto e no longo prazo e aos MACE. Além disso, a NLR tardia aumenta a capacidade da NLR na admissão de eventos adversos nesses pacientes. Até onde sabemos, esta foi a primeira vez em que a NLR tardia foi avaliada consistentemente nesse cenário.

A distribuição normal da NLR ainda exige debates. Forget et al.,^5^ estudaram indivíduos saudáveis, e os valores variaram entre 0,78 e 3,5, permanecendo estáveis após 48 horas. Recentemente, Kim et al.,^6^ observaram que a NLR aumenta ao longo do tempo em indivíduos com doença cardiovascular, atingindo os valores de pico próximo ao momento de um evento adverso. Um estudo recente demonstrou que os pacientes que experimentaram resultados adversos durante o período de acompanhamento tiveram um aumento agudo dos valores de NLR 48 horas após o procedimento.^4^ Esses resultados corroboram os achados de Kim et al.,^6^ descritos acima.

No presente estudo, quando os valores de NLR tardia foram usados para avaliar a capacidade de prever eventos adversos, houve um aumento significativo na AUC quando comparada à NLR na admissão. Isso pode ser explicado porque os neutrófilos são os primeiros leucócitos a infiltrarem o miocárdio infartado, liberando uma variedade enzimas proteolíticas que causam ruptura de placa, expansão do infarto, ativação da via da coagulação, e instabilidade elétrica cardíaca.^7^^–^^9^ Além disso, há evidências do prolongamento da vida de neutrófilos em placas instáveis.^10^ Em contraste com o aumento de neutrófilos nas áreas lesionadas do miocárdio, houve redução dos linfócitos devido ao aumento dos níveis de cortisol, catecolaminas e citocinas pró-inflamatórias no IAMCSST.^11^^,^^12^ Isso sugere que a resposta inflamatória exacerbada após o evento define os piores resultados para esses pacientes.

Na prática clínica da maioria dos centros mundiais, a contagem de leucócitos é realizada rotineiramente durante a hospitalização por evento coronário agudo. No presente estudo, uma medição de NLR no período de 48 a 72 horas foi um preditor forte dos resultados adversos, o que destaca a possível aplicação desses marcadores inflamatórios acessíveis e prontamente disponíveis para a estratificação de risco após o infarto do miocárdio.
