# Supplementary material for: Comparação entre a Relação Neutrófilo-Linfócito Precoce e Tardia na Predição de Eventos Adversos em Pacientes com IAMCSST submetidos à ICP Primária
Source: Arq Bras Cardiol. 2021 Mar 3;116(3):504–6. [Article in Portuguese] doi: 10.36660/abc.20200327 (PMC8159548; doi:10.36660/abc.20200327)
Supplement: Translation

# Translation: Early vs. Late Neutrophil-To-Lymphocyte Ratio for the Prediction of Adverse Outcomes in Patients with STEMI Undergoing Primary PCI

**Authors:** Guilherme Pinheiro Machado, Gustavo Neves de Araujo, Daniele Maltauro, Julia Custodio, Victoria Milan, Marco Wainstein

**Affiliations:** 1 Porto AlegreRS Brazil Universidade Federal do Rio Grande do Sul, Porto Alegre, RS - Brazil; 2 Porto AlegreRS Brazil Hospital de Clínicas de Porto Alegre, Porto Alegre, RS - Brazil; 3 Porto AlegreRS Brazil Universidade Federal de Ciências da Saúde de Porto Alegre Faculdade de Medicina, Porto Alegre, RS – Brazil

**Keywords:** Myocardial Infarction, Angioplasty, Mortality, Neutrophil-to-lymphocyte Ratio, Inflammation, Risk Factors

## Introduction

Admission neutrophil-to-lymphocyte ratio (NLR) has proven to predict adverse events in patients with ST-elevation Myocardial Infarction (STEMI).^1^^–^^3^ New evidence has shown that NLR continues to increase within 48-72 hours in patients who develop worse outcomes.^4^ Therefore, this study sought to compare the prognostic capacity of admission and late NLR for adverse events in patients with STEMI undergoing primary percutaneous coronary intervention (pPCI).

## Methods

This was a prospective cohort study with consecutive patients admitted with STEMI, who underwent pPCI and were followed up for 12 months. NLR was calculated by dividing the neutrophil count by the lymphocyte count obtained from the same blood sample. NLR was assessed at admission and 48-72 hours post-procedure (late NLR), as a part of routine care. Other details about procedural information, data collection, clinical definitions, exclusion criteria, and ethical guidelines are described elsewhere.^2^ High NLR was defined as above upper tertile. Receiver operating characteristic (ROC) curve analysis was performed to calculate the area under the curve(AUC) for the occurrence of short and long-term mortality and major adverse cardiovascular events (MACE). Multivariate analysis was performed by Poisson robust regression to evaluate the independent predictive value of late NLR. For the multivariate model, risk factors that were univariate predictors (at p <0.05) were initially considered factors or covariates. C-statistic analyses were compared with the De Long test, while Kaplan–Meier methods were compared with log-rank tests, performed using the MedCalc Statistical Software version 14.8.1 (MedCalc Software, Ostend, Belgium). All remaining statistical analyses were conducted using SPSS Statistics for Windows, v.21.0. (IBM Corp., Armonk, NewYork, USA).

## Results

Between March 2011 and December 2018, 864 patients were admitted to our institution, diagnosed with STEMI, and 779 were included in the analysis. Mean age was 60.68 (±12), 66.4% were male, 62.1% had hypertension, and 24% had diabetes.

In a multivariate analysis, when adjusted by age, pain-to-door time, previous chronic kidney disease, previous myocardial infarction (MI), hypotension at admission, femoral access, fluoroscopy time, contrast volume thrombolysis in myocardial infarction (TIMI) score, left ventricle ejection fraction ≤40% before discharge, late NLR remained an independent predictor of in-hospital death, in-hospital MACE, and one-year mortality (relative risk[RR] = 14.9, 95% confidence interval [95% CI]= 3.4 - 80.35, p= 0.001; RR= 3.4, 95% CI= 1.2 – 9.1, p = 0.01; RR= 7.6, 95% CI= 2.9 – 26.1, p= 0.01, respectively). The use of late NLR increased significantly the AUC of in-hospital mortality from 0.55 to 0.84 (Sensitivity 81.2%, Specificity 75.6%, Positive Predictive Value 24.5, and Negative Predictive Value 97.7). Discriminative data of other outcomes are described in Figure 1. At the end of a one-year follow-up, the rate of death from any cause was 28.6% in the high late NLR group, hazard ratio [HR] = 3.07 (95% CI = 1.9 - 4.8); p < 0.0001; Figure 2).

**Figure 1:** Receiver operator characteristic (ROC) graph showing areas under the curve (AUC) of admission neutrophil–to–lymphocyte ratio (NLR) and late NLR for (A) in-hospital death, (B) in-hospital major cardiovascular outcomes (MACE), (C) one-year all-cause mortality, and (D) one-year MACE

**Figure 2:** Time-to-Event Curves for one-year all-cause mortality for late neutrophil–to–lymphocyte ratio (NLR). Event rates were calculated by means of Kaplan–Meier methods and compared with the use of the log-rank test

## Discussion

In this present cohort-based study of STEMI patients undergoing pPCI, late NLR was strongly associated with short and long-term mortality and MACE. Moreover, late NLR increased the admission NLR's prognostic capacity for adverse events in these patients. To the best of our knowledge, this was the first time late NLR was consistently evaluated in this setting.

Normal distribution of NLR is still a matter of debate. Forget et al.^5^ have studied healthy individuals and values ranged between 0.78 and 3.5, remaining stable after 48 hours. Recently, Kim et al.^6^ observed that NLR increases over time in individuals with cardiovascular disease, reaching peak values around the time of an adverse event. A recent study showed that patients who experienced adverse outcomes during follow-up had an acute increase in NLR values 48h after the procedure.^4^ These results support the findings of Kim et al.^6^ described above.

In the present study, when late NLR values were used to assess the ability to predict adverse events, there was a significant increase in AUC when compared to admission NLR. This might be explained because neutrophils are the first leukocytes to infiltrate the infarcted myocardium, releasing a variety of proteolytic enzymes which cause plaque rupture, infarct expansion, coagulation pathway activation, and cardiac electrical instability.^7^^–^^9^ In addition, there is evidence of the prolongation of the lifespan of neutrophils in unstable plaques.^10^ In contrast to increased neutrophils in the damaged myocardial area, lymphocytes decrease due to increased levels of cortisol, catecholamines, and proinflammatory cytokines in acute STEMI.^11^^,^^12^ This suggests that the exacerbated inflammatory response after the event defines the worse outcome for these patients.

In the clinical practice of most centers worldwide, white blood counts are routinely obtained during hospitalization for an acute coronary event. In the present study, a measurement of 48-72h NLR was a strong predictor of adverse outcomes, which highlights a potential application of this inexpensive and readily available inflammatory marker for risk stratification of post-myocardial infarction.
